# Supplementary material for: Disparities in Lung Cancer: miRNA Isoform Characterization in Lung Adenocarcinoma
Source: Cancers (Basel). 2022 Feb 2;14(3):773. doi: 10.3390/cancers14030773 (PMC8833952; doi:10.3390/cancers14030773)
Supplement: Supplementary file 1 [file cancers-14-00773-s001.zip › cancers-1555527-supp/Figures S1-S6.docx]

Disparities in Lung Cancer: miRNA Isoform Characterization in Lung Adenocarcinoma

Rosario Distefano, Giovanni Nigita, Patricia Le, Giulia Romano, Mario Acunzo and Patrick Nana-Sinkam


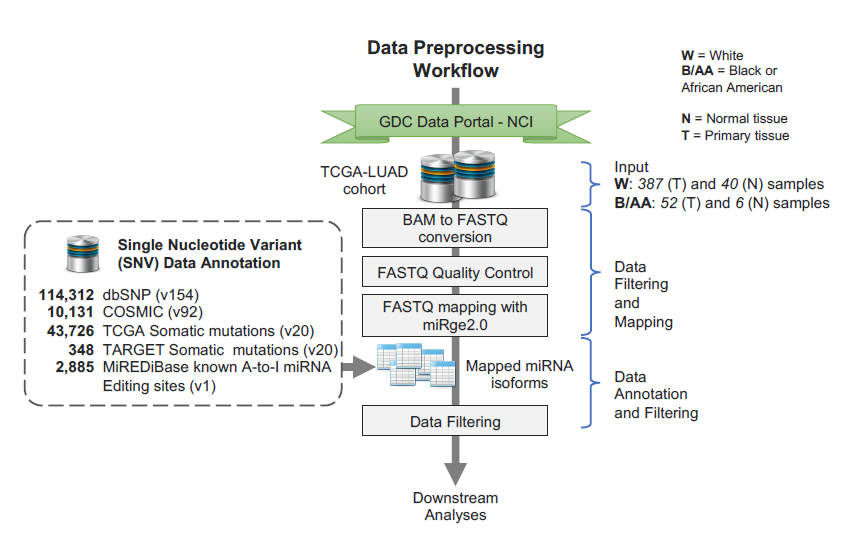


**Figure S1.** Data processing workflow of LUAD TCGA sRNA-seq samples.


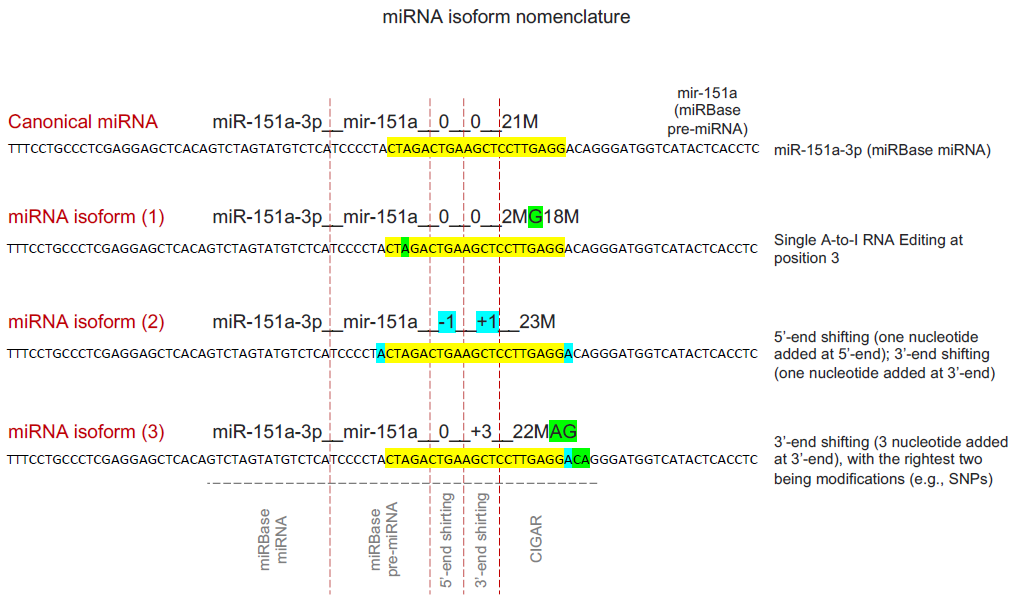


**Figure S2.** miRNA isoform nomenclature.


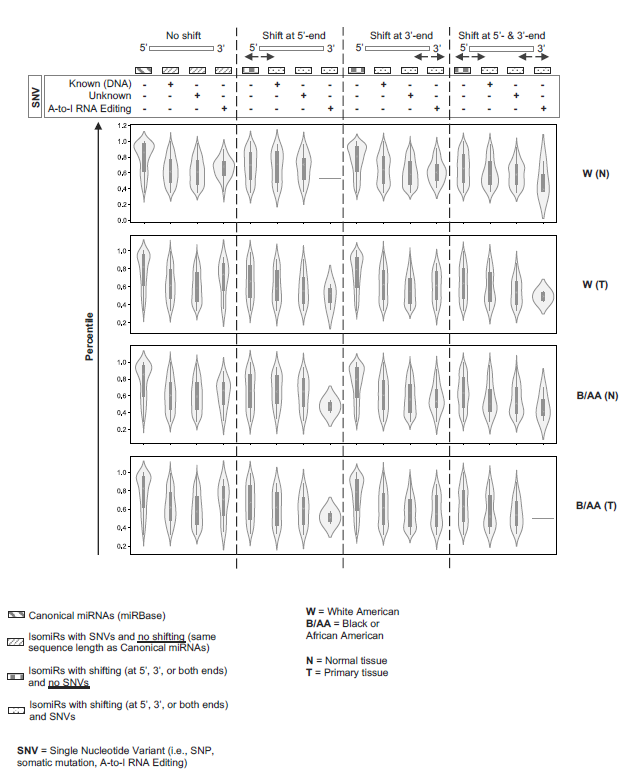


**Figure S3.** miRNA isoform expression distribution across the four group samples, namely W (N), W(T), B/AA (N), and B/AA (T).


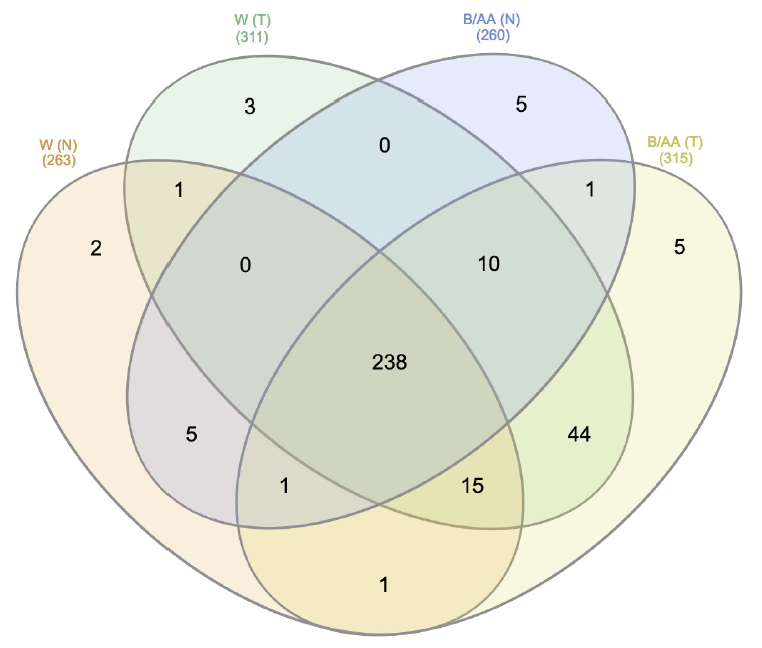


**Figure S4.** Expressed canonical miRNA in the four group samples.


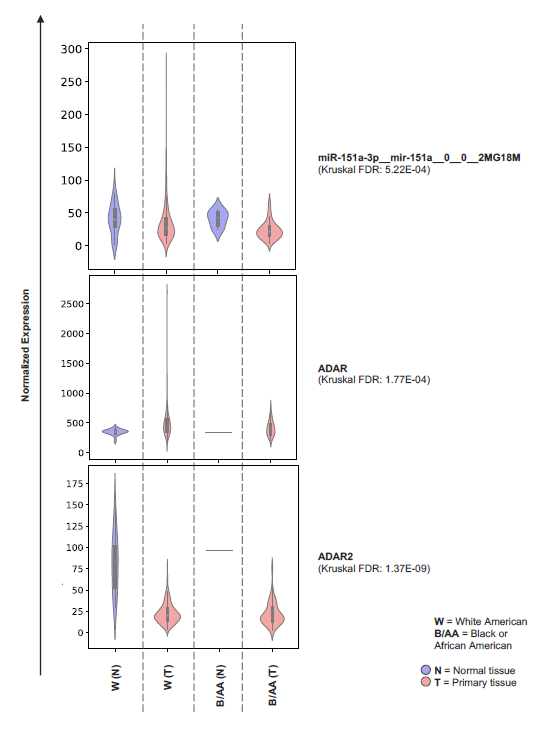


**Figure S5.** Comparison of expression between the edited miR-151a-3p and ADARs genes in LU-AD-TCGA data.


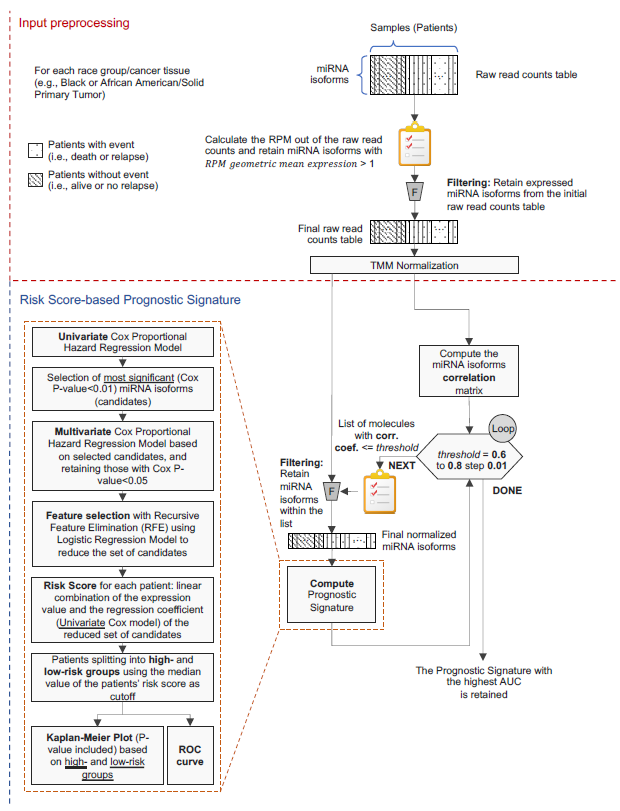


**Figure S6.** Two-stage workflow to identify prognostic miRNA isoform signatures
